# Clinical leadership development in the NHS – a study in urgent and emergency care (UEC)

**DOI:** 10.1108/JHOM-03-2024-0079

**Published:** 2025-03-14

**Authors:** Hein Scheffer

**Affiliations:** East of England Ambulance Service NHS Trust, Melbourn, UK

**Keywords:** Clinical, Leadership, Education, Development, Training, NHS

## Abstract

**Purpose:**

This paper explores why clinicians in an emergency department (ED) become leaders, their experiences of leadership and their future developmental needs. It focuses on emerging leaders, middle management, and senior management whilst addressing the knowledge gap in identifying the training needs of clinical leadership in urgent and emergency care (UEC).

**Design/methodology/approach:**

This study utilised both surveys (*n* = 36) and semi-structured interviews (*n* = 12). Qualitative data were analysed using descriptive statistics, whilst qualitative data were analysed using a thematic approach, drawing on a conceptual framework based on the inter-related concepts of culture, professional identity and leadership development. This paper focuses on the third concept and offers insights into the journey and challenges faced in making the transition from clinician to leader.

**Findings:**

The findings revealed that most clinical leaders received insufficient training to prepare them to be leaders in UEC.

**Research limitations/implications:**

This study was originally intended for a single English Acute Trust, rendering the data limiting, as an interpretivist study. The fact that three Trusts were used for the semi-structured interviews helped with the triangulation of data.

**Practical implications:**

The paper proposes an original leadership development framework for UEC to support leaders who are often excellent clinicians, to be equally brilliant and appropriately empowered leaders.

**Originality/value:**

A more individual-centric focus on clinical leadership development is advocated, offering an original leadership development framework to support leadership development and contributing to the wider literature on education.

## Introduction

While effective leadership, management and associated leadership development programmes are known to be crucial for the success of institutions (see Carmichael *et al*., 2011), it is surprising to note that the topic of leadership development has been overlooked and under-researched in the National Health Service (NHS) in England. This was highlighted by the fact that the concept of “talent management” only became a key point of debate within the NHS as late as 2004 (Powell *et al*., 2012). Since the creation of the NHS on Monday 5th July 1948, it took a further eight years until 1956 before it was recognised that there needed to be a structured process in place to ensure the development of leaders in the NHS (Begley, 2020). In addition, there have been a plethora of independent reviews over the past twenty years, all of which explored leadership development. However, the federated nature of the NHS does not make talent management easy, as it does not allow for across organisational and system-wide talent management. This remains an area that requires significant development and focus in the future.

The NHS is operating in an ever-increasing complex environment. The rise in public expectation and demand following the Covid pandemic and the workforce shortages in the NHS are all contributing factors that necessitate a well-developed and equipped leadership cadre to lead the NHS through these challenging times. One of the key issues within the NHS regarding leadership practice is that the majority of clinical leaders joined the service as clinicians and then find themselves taking on a leadership role, often without the core skills and experiences needed to enable them to lead efficiently, sustainably and thoughtfully (Darzi, 2008, 2024; Démeh and Rosengren, 2015; Fulop and Day, 2010; Ham *et al*., 2011; Klaber and Lee, 2011; Messenger, 2022). NHS managers today have very little support on their transitional journey, which was originally reflected on by Nicholson and West (1988), when they considered the meaning, impact and rationale of transition of managers in the late 1980s (Nicholson and West, 1988, p. 1).

Ham *et al.* (2011) contend that clinical leaders are often seen as “reluctant amateurs”, whilst the NHS offered little support for personal development in preparing clinical leaders for their leadership roles. Whilst there is emerging work being done in the NHS exploring leadership practice (see Ham, 2011; Kerr, 2018; Kings-Fund, 2011; Messenger, 2022; NHSE, 2016, 2019a; Powell *et al*., 2012), there appears to be very little work done previously in the NHS, conducting insider research by NHS leaders, exploring leadership development within the sector. This suggests that policymakers may not fully understand the nuanced and complex leadership development needs or activities that are required at the organisational level, as has been found in similar research in the higher education sector (Floyd *et al*., 2023), ensuring compassionate, effective and engaged leadership (Messenger, 2022; West and Bailey, 2019). Therefore, the aim of this paper is to address the knowledge gap by drawing on the experiences of emerging leaders, middle management, and senior management in urgent and emergency care, exploring their journey in becoming leaders in the emergency department (ED) of an English Acute NHS Trust and their development needs for the future.

Following the analysis of this study and drawing on the inter-related concepts of culture, professional identity and leadership development, a leadership development framework is proposed based on an individual centric model of leadership development for organisations to consider. This framework offers an original contribution to our knowledge in this area, building on the principles of work role transition theory (Nicholson, 1984).

The importance of this paper is founded on the need to train, educate and develop NHS managers and leaders in the art of leading others and the core principles of management, as the area of “keen amateurs” (Ham, 2011; Ham *et al*., 2011) is outdated and not what is required for a sustainable NHS in the future.

Following the introduction, this paper is presented in four overarching sections. Firstly, it considers the **literature review**; secondly it describes the **methods** used, and thirdly it offers the **results and discussion** which includes the proposed individual centric leadership development framework, followed fourthly by a sample **leadership development model**.

## Literature review

### Introduction

This article examines the journey that clinical leaders undertake to become managers and leaders, reflecting on their training (or lack thereof), the support they need and the transition process, as defined in models like the “work role transition” (Harris *et al*., 2012; Nicholson, 1984; Nicholson and West, 1988). A thorough literature review was conducted, reviewing over 400 sources and identifying key themes such as culture, professional identity and leadership development.

### Organisational culture, values and behaviours

Dixon-Woods *et al*. (2013) highlight that while healthcare organisations aim to provide high-quality care, shortcomings in organisational culture persist. They distinguish between “problem sensing” and “comfort seeking” leaders, with the latter fostering environments where frontline staff are unfairly blamed (Dixon-Woods *et al*., 2013). Schein (2004) argues that culture, shaped by shared assumptions and values, is heavily influenced by leadership. Leaders act as “parent figures,” shaping collaboration in complex organisations (Schein, 2004).

Nicholson’s (1984) “work role transition” describes major career changes, including shifts to leadership roles, as unpredictable and often lacking planning. Roberts (2005) asserts that leaders must consciously inspire constructive behaviours to improve productivity and accountability. He warns against superficial changes, such as headcount reductions, citing examples where poor strategies harmed productivity. Castro and Martins (2010) and Eustace and Martins (2014) link organisational climate to job satisfaction, distinguishing culture as foundational to values and climate as the atmosphere shaped by structures and leadership behaviours (Eustace and Martins, 2014). The reality is that the ever-increasing need for scrutiny in the public sector also requires a higher degree of professionalism, which is achieved through structured high-quality leadership learning that, at the time, was just not available (Ham *et al*., 2011).

Grint (2010) categorises problems into tame, wicked and critical. He notes that leaders who excel in crises situations may become overconfident and untouchable. Traditionally good managers and leaders are often ignored, as there is no “crises” under their watch, but charismatic leaders, who thrive in a crisis situation could artificially present any challenge as a crisis, with disastrous effects to their followers (Grint, 2010, pp. 169–185).

As the NHS has been slow to adopt talent management principles (Powell *et al*., 2012), it is not surprising that clinical leaders are described as “keen amateurs” (Ham *et al*., 2011). They are mostly individuals who are clinicians first, and managers second who are commonly not well transitioned into their managerial role (Fulop and Day, 2010) and whilst progress had been made, more is required (see Dekker *et al*., 2022; Hougaard *et al*., 2022; Messenger, 2022; West, 2021).

### Professionalism and professional identity

According to the American Board of Internal Medicine (American Board of Internal Medicine, 2011) “Professionalism in medicine requires the physician to serve the interests of the patients above his or her self-interest” (p. 5). They went further in defining the “core of professionalism” as constituting those attitudes and behaviours that serves to maintain an altruism approach to patient care with the following specific core elements that forms the basis of professionalism to include (1) altruism, (2) accountability, (3) excellence, (4) duty, (5) honour and integrity and (6) respect for others (p 5–6).

The Royal College of Physicians (RCP) established a working group in 2005 to explore “the nature and role of medical professionalism in modern society” (RCP, 2005). The RCP defined professionalism as:

a compilation of attitudes, values, behaviours, and relationships that supports the public’s trust in doctors (RCP, p. 45).

Interestingly, neither the NHS guidelines for “Maintaining High Professional Standards” (DoH, 2003), which governs the professional standards and practices expected of clinicians in the NHS, nor NHS Employers’ toolkit on “Professionalism and Cultural Transformation” (NHSE, 2019c) contains a succinct definition of what is meant by “professionalism”, which underpins the RCP’s concerns about the “lack of research on “professionalism” in the professions [they] regulate” (RCP, 2005, p. 1). Despite NHS efforts, a clear definition of professionalism is lacking, raising concerns about the alignment of workforce practices with professional goals or behaviours (NHSE, 2019b).

Inter-professional education and collaboration, as explored by Floyd and Morrison (2014), highlight the complexities of shared professional identities. Wenger’s “community of practice” (2006) further underscores the value of informal learning in fostering collaboration and improving outcomes.

### Leadership development

Neuroscience shows the brain’s adaptability supports lifelong learning (Blackman, 2014). Yet, leadership remains elusive to define, evolving through experience and supported learning. Ham *et al*. (2011) found that clinicians transitioning to leadership roles often lacked formal training, relying on self-directed learning and experience (Ham *et al*., 2011). Grint (2010) categorises problems as tame, wicked or critical, cautioning against leaders who create crises to showcase their charisma (Grint, 2010).

Bolden *et al*. (2003) conducted a detailed review of leadership theory and competency frameworks, exploring McGregor’s Theory X and Theory Y managers, Blake and Mouton’s Managerial Grid and Fielder’s Contingency model, to mention a few (Bolden *et al*., 2003). The NHS has invested significant resources over the past 13 years in creating 12 leadership development and competency frameworks, whilst no less than 12 independent reviews have been published relating to leadership development, between 2008–2023, yet the evidence in this article still suggest that there is not sufficient real-term development being done to make a meaningful difference in the preparation of clinical staff in becoming managers or leaders.

Structured leadership programs will address these gaps, focusing on the core principles of management, fostering distributed leadership and enabling individuals to improve patient care. Al Ariss *et al.* (2014) emphasise the importance of technology and diversity in shaping future leadership and talent management strategies (Al Ariss, Cascio and Paauwe, 2014).

### Conclusion

Theoretical models such as Nicholson’s (1984) Schein’s (2004) and as was reviewed in detail by Bolden *et al*. (2003) offer insights into leadership learning and role transition. While the NHS has developed numerous frameworks, structured programs must better support clinicians transitioning to leadership roles, promoting diversity and leveraging technology. Continuous leadership development, grounded in empirical evidence, is essential for delivering compassionate, high-quality care.

## Methods

Hegelund (2005) contends that a mixed-method approach has much to offer (Hegelund, 2005, p. 663). This combination of both qualitative and quantitative research offered the best of both worlds (Flick, 1998) and was thus used in this study. This included one-to-one interviews and focus groups in terms of the qualitative paradigm (Kvale, 2007); and surveys, descriptive, experimental or correlational research in terms of the quantitative paradigm (Ercikan and Roth, 2006).

Onwuegbuzie and Leech (2004) suggest that a dual methodology of both qualitative and quantitative research creates “a bridge” between the schism of the two methodologies, offering an obvious potential for researchers (Onwuegbuzie and Leech, 2004).

### Settings

This study focused on clinical leaders in the ED only, of an acute Trust in England, as the ED is often seen as the front-door of the hospital. This provided insight into the experiences, perceptions and realities of these clinicians who hold leadership roles, on their journey from being clinicians to becoming clinical leaders. It further explores the development and education they have received, wanted to receive, or needed, to ensure they are effective clinical leaders.

This paper explores the perceptions and impact of three levels of clinical leaders, based on the NHS job-banding structure, titled *Agenda for Change (AfC)*. For ease of reference, these are defined as:

1. **Emerging leader** ∼ an individual who shows high potential for future development, currently in AfC band 4–6, including someone on a junior doctor’s contract.
2. **Middle management** ∼ team leaders or intermediate leaders who are on a career path towards senior management, traditionally in AfC band 7 – 8C, or medical and dental (M&D) staff grades.
3. **Senior management** ∼ the highest level of management, or positions just below the Board, traditionally in AfC bands 8D, 9, or M&D consultant contract.

### Study instrument and data collection

In stage one of data collection, a survey with 31 questions was developed, which were originally sent to a sample population of 235 clinical leaders within the ED of one English NHS Trust. The composition of these staffing groups was:

1. **30** emergency medical technicians,
2. **149** registered nurses and
3. **56** medical and dental staff.

The survey was developed from the literature review initially, resulting in 70 questions, which were refined through a process of iteration and elimination resulting in the final 31 questions. Eight versions were tested with the help of a focus group, aiming to gain a broader insight into the applicability of the survey questions. Consideration was given to Freitas *et al.* (1998) who cautioned against the researcher’s own biases, as focus group moderator, which could impact the data negatively (Freitas *et al*., 1998).

Following the conclusion of the survey phase of this study, 12 participants identified themselves as being willing to be interviewed. All 12 were written to, with the aim of planning the interview that form the “analysis continuum” of Crowe *et al.* (2015). It was this “analysis continuum” (Crowe *et al.*, 2015) that was used in this study, utilising both thematic analysis (TA) and content analysis (CA), the process of analysis differs. Braun and Clark (2006), as referenced by Crowe *et al.* (2015), describe this analysis continuum as the “theoretical flexible method” of data analysis as follows:

1. **Thematic Analysis** (TA)
  - **Latent** ∼ present but not immediately visible, needs exploration to identify;
  - **Interpretive** ∼ requires deductive skills to make sense of its meaning;
  - **Inductive** ∼ forming a point of view by reaching conclusions, making judgements and inferences from a given set of facts;
  - **Inability to be calculated** ∼ does not lends itself to calculation, views are determined by facts, categorisation and cannot arrived upon by means of numerical calculation.
2. **Content Analysis** (CA)
  - **Manifest** ∼ data that is readily visible, evident or obvious following some review;
  - **Descriptive** ∼ data is explained in terms of its characteristics that gives meaning to it;
  - **Deductive** ∼ a process of logical assumptions that defines a logical conclusion;
  - **Ability to be calculated** ∼ the results can be achieved by applying a computed calculation (Braun and Clark, 2006 in Crowe *et al.*, 2015).

The final question of the survey invited participants to volunteer for the semi-structured interviews. Although the survey was totally confidential and participants could only be identified in terms of how they declared their own sex, pseudonyms were allocated to all participants using the alphabet and a “name-book” (Astoria, 1997) for ideas of pseudonym names.

### Study sample

Following the distribution of the survey, **36** participants returned their responses, resulting in a response rate of 31%.

In establishing the one-to-one semi-structured interviews, **12** participants, who operated in multi-disciplinary teams (MDTs) from other EDs than those participating in the survey were identified, representing 6 middle management and 6 senior managers.

Regrettably none of the emerging leaders originally approached were willing to participate in the semi-structured interviews. According to Spehar *et al*. (2012), this could be due to the fact that clinicians did not always anticipate a career in management, did not identify as being leaders, or joined the healthcare sector to help others as clinicians first and foremost (Spehar *et al*., 2012).

Due to Covid restrictions and the challenges faced by the NHS at the time, all semi-structured interviews were conducted with participants via MS Teams. Some of these interviews had to be re-scheduled several times, around the operational availability of participants.

A fact sheet was provided in advance to both survey and semi-structured interview participants, confirming the purpose of the research that their personal information would be treated in confidence, and that their participation was fully voluntary. Participants were free to withdraw at any stage of the process.

### Ethical approval and consent to participate

Standard ethical principles were followed during this study. Following receipt of the fact sheet, participants also confirmed that by completing the survey they gave their consent for their responses to be used for the purpose of research.

From an NHS perspective, the Information Officer of the Medical Research Council (MRC) confirmed that as this study only involve NHS staff, and as no member of the public was involved in this research, no additional ethical approval was needed.

## Results and discussion

Following the literature review, surveys and semi-structured interviews, whilst applying inductive methodology, the first key theme that emerged from the analysis was that of organisational culture.

### Organisational culture

It became clear that within this study, there were different perceptions of organisation culture, although it was acknowledged that this would vary from trust to trust. It was suggested that there was a lack of understanding about the working of the ED, whilst external factors play a key role in organisational culture (see Table 1).

The perception on culture may be different from the perspective of the Trust Senior Management. This is because they do not understand the pressures that we face. They just look at the targets, pressures at the back door and the reality that we must keep on admitting patients through the front door. ∼ Victor, ED Consultants (Senior management).

There are external factors that influence the culture in the department, like training or system problems, that people are aware of, but it’s often beyond our control. These factors have the greatest adverse impact. This is not an excuse, but culture is impacted by a variety of elements, and it’s often factors like the availability of the right levels of staff, training, or time that makes performance management difficult. You have to deal with what you have. ∼ Noran, ED Consultant (Senior management).

**Table 1:** Culture in the emergency department [Q6]

	Emerging leaders		Middle management		Senior management		Total	
How would you describe the emergency Department’s culture? [Q6]								
	*n*	%	*N*	%	*n*	%	*n*	%
Collaborative	2	5.6%	3	8.3%	1	2.8%	6	17%
Collegial	2	5.6%	0	0%	2	5.6%	4	11%
Democratic	4	11.1%	0	0%	1	2.8%	5	14%
Hierarchical	7	19.4%	10	27.7%	2	5.6%	19	53%
Other	0	0%	0	0%	2	5.6%	2	6%
Total	*15*	*41.6%*	*13*	*36.1%*	*8*	*22.2%*	*36*	*100%*

**Source(s):** Author’s work

In exploring the circumstances that are likely to impact the perceptions on culture in the ED, it was acknowledged that the perceived culture would be different for different people and will vary in different ED’s because of the lack of “*support and empathy from the senior team*” and the “*…time pressures, understaffing, different skill mixes, progress chasers and a broken NHS system”*. – Feline (Emerging Leader) Q11a. This finding is supported by the studies of Yiing and Ahmad (2009) and Marmaya *et al.* (2010) who concluded that supportive leadership who demonstrated their support through their conduct, commitment and behaviour played an influencing role on the ultimate experience of staff when it comes to perceived culture in an organisation. Contrary to their findings, an emerging leader articulated their frustrations:

Managing patients’ expectations compared to reality, working while understaffed so doing the work of multiple people, disagreeing with decisions from higher up but having little authority to challenging them is very demoralising. ∼ Carla (Emerging Leader) Q11a.

Although the majority, **19** [53%], of survey participants felt that the culture in ED was perceived as hierarchal, from the feedback in the semi-structured interviews, the views were very spread. Three of the twelve [25%] felt that the ED has a “*massive blame culture, with a strong focus on performance targets*”; whilst Luann, ED Consultant (Senior Management), suggested that there is a duality to cultural perception, one being the culture in the Trust and the other that of the ED. He confirmed that in his perception the Trust culture is “*dictatorial*” with a strong focus on “*hierarchical leadership*” whilst in the ED the culture was described as “*not dictatorial, but more of an anarchy*”. Luann suggested, similar to that of the senior management who participated in the surveys, that leadership styles in the department must change according to issues and work pressures in the ED. This “anarchy” being explained as a perpetual state of disorder (OCD, 2008, p. 31), not because ED is unorganised, but because it is so busy – (Quiana, ED Matron).

However, it has been suggested (see Dekker *et al*., 2022; Oliver and Memmott, 2006; West *et al*., 2015) that culture is more than hierarchy; it is about engagement with everyone in the group, team or department. West *et al*. (2015) contend that culture is not something that stands separate, but is created and realised, by the people within (West and Bailey, 2019).

Nicholson and West (1988) previously reflected on organisational structure and suggested that they are “not pyramids” but described them as “scattered encampments on a wide terrain of hills and valleys” and on career transition and progression they suggested that careers are not “ladders, but stories about journeys and routes through and between the encampments” (Nicholson and West, 1988, p. 94).

It is therefore conceivable that culture cannot be changed through the writing of a culture strategy, or a developmental programme, or the appointment of a culture lead. Rather, culture is created by people’s behaviours, through compassionate engagement, whilst holding people to account for operational delivery across all the respective levels of an organisation (West and Bailey, 2019). It is the change in behaviour that manifest in culture change.

Culture in the ED has also been explained as the level of “psychological safety” which “is about candour and willingness to engage in productive conflict”. To ensure various perspectives are heard, creating space for innovation and education (Edmondson, 2003, 2019; Lencioni, 2002) that is visible and being practiced within the Trust.

Considering the workforce challenges across the NHS, it will be key to ensure that international recruitment into the NHS remains viable, sustainable, cost effective and does not equate to a “leaking bucket”, with the service having a continued revolving door of international nursing and/or medical staff coming and going on an endless basis. The principles of transition theory are worth considering, in future development programs, whilst the NHS will do well in considering these principles in supporting managers through job role transition (Nicholson, 1984; Nicholson and West, 1988) in becoming leaders. Alternatively, and likely more importantly, developmental strategies in growing our own (Oliver and Memmott, 2006) should be considered.

### Professional identity

The analysis suggests that professional identity as a concept also plays a significant role in leadership and therefore will impact leadership development as well. Participants talked about their own individual identity, influenced by cultural and religious customs, professional identity, and a lack of a sense of belonging, compounded by bullying behaviours based on their ethnic identity. Some participants indicated a perception that there was a divide between nursing and medical staff or the directorate management, whilst the counter was also argued, in suggesting that everyone worked well together. Some respondents also cited an affiliation to a specific clinical identity.

I think there is a divide, but the divide is not between the nursing or the medical staff. It is in the behaviours of the directorate management, the directorate clinicians and nursing staff who are caring for patients, that is where the divide is. ∼ Luann, ED Consultant (Senior management).

I think there is certainly a divide, but the primary identity as a doctor, is adhering to professional standards. Then choice of speciality and place of work become relevant as secondary. ∼ Xandria, Middle management (Q14.a).

I don’t think we do have a strong divide at all. I think we work very well together; we are all striving to do the right thing for the patients. ∼ Sancia, ED Consultant (Senior management).

As professional identity was a strong contributor to how professionals make sense of what they do, this ethical framework has been described as a structure of honourable values according to which people live their lives.

Unfortunately, within the health sector, there remains a reluctance to embrace this change, as professions tend to be very protective of their vocations, in their belief that doctors should only do what doctors were trained to do; nurses should do what nurses were trained to do, and only paramedics or allied health professionals (AHP) can do what paramedics or AHPs were trained to do, etc. This was confirmed by Ireland (2023) reporting on the confusion caused by Physician Associates (PA’s) and the call that they should not be regulated by the General Medical Council (GMC), but a separate regulatory body. He referenced a clinician who have said “This is about our profession; this is about our future”. ‘It’s time to say, “I worked bloody hard for my MB ChB and nobody is taking that away from me’” (Ireland, 2023).

This professional, but narrow view, is out of step with the reality that there are just not sufficient clinical staff available, world-wide, and as such new ways of working, multi-disciplinary team working, and collaborative co-designing of creative new clinical and non-clinical roles should be considered to fill the gap. This will enhance rather than devalue the healthcare profession.

In terms of professional behaviour, from the survey data, the perception of a rift between the professional behaviours of medical and nursing staff, had a 50/50 divide, and two participants who were not sure either way. In this regard, it was reported that some clinical leaders have seen a significant change since they have stepped into a clinical leadership role, in that they were recognised more, they got results quicker, or people listen to them more.

If I write to other trusts, as a consultant, I think I get more of a response now rather than five years ago, before I became a consultant. ∼ Luann, ED Consultant (Senior management).

Male consultants generally get things done quicker, i.e. their bloods are done much more prompt than their female counterparts, due to the institutional memory. The trust respect, remember and respond to the male consultants much better than to the female consultants. I have taken over from my male predecessor several years ago, but they still write his name on the requisition. As if he requested the bloods, or tests. So, the organisational memory takes a really long time to change, especially when past personalities have been fairly dominating. ∼ Oriana, ED Consultant (Senior management).

But the same amount of people argued that for them there was no difference; they were there for the patient first and foremost and they did not believe that there was much of a difference between the various clinical disciplines in the ED, as the nature of the department requires of everyone to play their part, working as a team for the benefit of the patient.

Being a leader does not change how I see myself. I remain foremost a nurse. However, I also believe that everyone has leadership abilities. I have a band 3 who does an amazing job in her field, and as far as I’m concerned, she leads the way in her area of responsibility. The role modelling from her is no different, because she’s a band three, to me as lead nurse. ∼ Tara, Lead Nurse in ED (Middle management).

It was these challenges that impact professional identity for some, and whilst most felt there was no divide between the clinical professions or real challenges within the ED, it was acknowledged that the ethical dilemmas created between their organisational positions, in terms of power and politics, and their regulatory bodies did lead to conflict between clinical leaders. What was challenged is the lack of clear career pathways and developmental support, for clinicians who are on their journey of being clinical leaders.

Nobody really sits you down as a consultant and help you plan your next steps as a clinical leader. I think this should start much earlier and maybe someone should sit down with new consultants and help co-design their pathway, it’s not clear or easy at the moment. ∼ Sancia, ED Consultant (Senior management).

Professional identity is nearly as complex as organisational culture. But what was clear from this study is that professional identity is defined by a variety of elements including how professionals make sense of what they do. In addition, and considering that neither the NHS guidance on “Maintaining High Professional Standards” (MHPS) nor NHS Employer’s toolkit on culture defines “professionalism” as a concept, the question remains, whether clinicians are truly taught, during their forming years, what “professionalism” and/or “professional behaviours” means for them, or what may be expected of future clinicians and clinical leaders in this regard.

### Leadership education and development

The analysis suggests, as has been found elsewhere (see Ham *et al*., 2011), that participants believed that they did not become clinicians to became leaders. They felt that they were not sufficiently supported on their journey of transition (Harris *et al*., 2012; Nicholson, 1984; Nicholson and West, 1988) or the basic elements of management or leadership. Nor were they prepared how to lead others, with compassion, inspiration and support, to lead and develop those in their charge (Sinek, 2009).

The key challenges for clinical leaders were the lack of capacity, bespoke individual development and availability of time. They articulated personal developmental needs, to have the opportunity to develop and grow, but due to the persistent pressures on time, abstractions, and challenges facing the NHS, like capacity and time to develop was just not possible.

In this regard, it was noted that **12** [33.3%] participants indicated that they have not received any training for their current role at all. It was confirmed that this group, received their normal “mandatory training” as is required in the NHS, but no additional training or development was reported. Eleven [30.6%] participants confirmed that they had “little training” in preparation for their current role; **8** [22.2%] indicated that they had a moderate amount of training, whilst **5** [13.9%] confirmed that they had received “a great deal of training”. From this final group, it was not clear what the extend or level of training was that they had received. Contrary to most, some argued that there was sufficient training available to choose from, as one ED consultant submitted.

Training and development opportunities is not the problem. There is a long list of training programmes to choose from, but people do not have the time to attend. Availability of time remains a challenge. We must truly protect time for clinical development. ∼ Sancia, ED Consultant (Senior management).

When enquiring how useful the training was that had been received, **9** [25%] indicated that their training was “extremely useful”; **8** [22.2%] indicated it was “slightly useful”; **7** [19.4%] confirmed their training was “quite useful”; **6** [16.7%] indicated that the training they had received was “not useful at all”; with **4** [11.1%] confirming that their training was “moderately useful”; and finally, **2** [5.6%] participants selected “other” and indicated that they either received “no training” at all, or “none other than mandatory training” respectively. An ED matron felt strongly that clinical leaders are not sufficiently prepared for their roles.

Clinical leaders are not effectively equipped, it’s like “here you go, be a clinical leader” but originally our curriculum did not prepare us for this ∼ Quiana, ED Matron (Middle management).

In the quantitative data from the survey, **12** [33.3%] participants submitted that they received no formal education or development from their trust, other than the traditional mandatory training. These consist of several statutory e-learning modules that all NHS staff must comply with, to ensure they remain fit to practice. Some examples of these are information governance; adult safeguarding; child protection; equality, diversity, and inclusivity, to mention a few. It is this sense of the “keen amateurs” referenced by Ham *et al.* (2011) when he challenged the NHS as a system, in that it offered little structure in terms of personal and professional development and preparing clinical leaders for the demanding role of management. These individual development needs could be addressed by Wenger’s (2006) concept of “community of practice” (CoP) wherein he proposes an environment of informal learning (Li *et al*., 2009). “A useful perspective on knowing and learning” (Wenger, 2006). In exploring why clinicians became clinical leaders, the following was noted.

From Table 2, it was noted that **19** [52.8%] confirmed that they “wanted a new challenge” and that this level of leadership was their natural next step; **18** [50%] indicated that becoming a clinical leader was part of their “personal development plan” (PDP); **16** [44.4%] confirmed that they were “asked to take on more responsibility”; **13** [36.1%] indicated that they “enjoyed developing other people’s careers”.

**Table 2:** Rational for becoming a clinical leader [Q16]

			Emerging leaders		Middle management		Senior management	
Why did you move from a clinician to a managerial or leadership role? [Q16]								
	*n*	%	*n*	%	*n*	%	*n*	%
The longest serving in the department	3	8.3%	2	5.6%	1	2.8%	0	0%
Enjoy developing other people’s careers	13	36.1%	6	16.7%	4	11.1%	3	3.8%
Always wanted to lead others	12	33.3%	5	13.9%	5	13.9%	2	5.6%
Wanted a new challenge	19	52.8%	8	22.2%	8	22.2%	3	8.3%
Wanted to make a wider impact within the Trust/ED	12	33.3%	6	16.7%	2	5.6%%	4	11.1%
Wanted to move away from operational delivery	3	8.3%	2	5.6%	1	2.8%	0	0%
Was asked to take on more responsibility	16	44.4%	9	25%	4	11.1%	3	8.3%
A natural division of responsibility	12	33.3%	7	19.4%	3	3.8%	2	5.6%
Part of my personal development plan	18	50.0%	9	25%	6	16.7%	3	8.3%
Other	6	16.7%	1	2.8%	2	5.6%	3	8.3%
*Number of answers from 36 respondents*	*114*		*55*		*36*		*23*	

**Source(s):** Author’s work

Three groups of **12** participants, or 33.3%, indicated that (1) it was “a natural division of responsibility”; (2) they always “wanted to lead others”; and (3) they wanted to “make a wider impact on the Department or Trust”, respectively.

These reasons for becoming a clinical leader were critically challenged by a participant in the semi-structured interviews, who indicated that she had no intention to progress as a clinical leader.

Becoming a leader in ED is a bit like politicians. They think they go into it because they think they can make a difference, and they can do a really good job. But the reality is that you end up not having much influence and eventually becomes just like them. ∼ Karin, ED Nurse Practitioner (Middle management).

She argued that leaders lost so much of their own individuality, as they had to leave too much of who they are, in favour of being a “corporate being”. Karin submitted that whilst many leaders start with the best intentions, they end like all the others, they don’t truly change the behaviours and practices within the ED. Instead, clinical leaders are changed by the practices in the ED as they adopt more mediocre approaches. Karen felt that “managers very quickly forget how it was when they ran the floor”, she observed that “managers become managers, because they think they could be the manager they always wanted” ∼ Karin, ED Nurse Practitioner (middle management). Another interview participant confirmed that there is a need for a variety of approaches, in submitting:

I don’t think we had equipped our clinical leaders enough as it’s almost like we just expected them to naturally be able to learn how to manage a group of very different specialities, when the interactions between different specialities, and different groups of people, are very different. ∼ Uriah, Managing Director (Senior management).

The rationale for clinicians becoming clinical leaders varied as some suggested their progression was “planless” (Nicholson and West, 1988). Regardless, all recognised that some core competencies for leadership development would be essential; they lacked development; they lacked the basic principles of management and leadership and there is little alignment between the different programmes currently available or addressing individual-centric needs.

Nicholson and West (1988) found that “it was not unusual to find people feeling simultaneously plateaued but satisfied, self-directed but planless, externally directed but orderly in their career type” (Nicholson and West, 1988, p. 93).

### A proposed framework to develop individual centric leadership development

**Figure 1:**
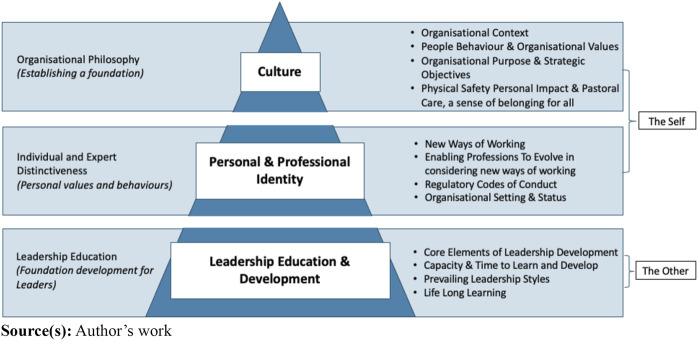
Leadership development framework

From the analysis of the data a leadership development framework is proposed in Figure 1, which forms the departure point for organisations to develop an individual centric approach to leadership development for effective, compassionate and sustainable leadership for the future. The creation of the proposed leadership development framework within a supportive working environment is essential for growing leaders of the future (Kazmi and Naaranoja, 2015). It was also argued that a “one-model-fit-all” approach to cover the developmental needs of senior leaders, middle management and emerging leaders, in a historical traditional way is inappropriate, as different groups and individuals have different needs (Castro and Martins, 2010; Eustace and Martins, 2014).

It was suggested that the NHS showed little interest in clinicians’ career development, as there is a stronger focus on operational delivery and targets, resulting clinical leaders to be left to their own creative resources in building their skills in terms of on-the-job development using the practice of “see one, do one, teach one” (Ham *et al*., 2011, p. 16). This approach does not take cognisance of the individual’s personal developmental journey, strengths and developmental needs. Neither does it consider the predictor variables of “work role transition” (Nicholson, 1984; Nicholson and West, 1988).

The original contribution offered by this study is based on the inter-related concepts of culture, professional identity and leadership development, which forms the final elements of a proposed clinical leadership development framework as defined in Figure 1.

**Figure 2:**
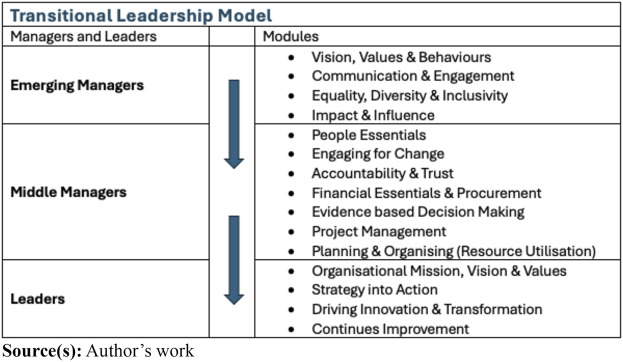
Sample of a transitional leadership development model

It was recognised that there are many leadership development programmes currently in place and available for use across the NHS. This study offers a leadership development framework, which provides greater flexibility, based on the needs of the individual in relation to the organisational context and requirements, considering culture, personal and professional identity, and leadership development in Figure 1. Rather than having a single leadership development model or programme, that is imposed on all clinical leaders, the proposed leadership development framework suggests an individual centric modular approach. Thus, bridging the gap between clinicians and clinical managers and leaders. The development program that flows from this framework should make distinction between managers and leaders, as the one focus on the utilisation of resources, delivering operational outcomes, whilst the other focus on future thinking and strategic vision, delivering organisational strategic objectives and continues improvement to the benefit of both the individual and the organisation. Based on the feedback from participants, a sample of what could emerge as a development model is set out in Figure 2.

It is not possible to create a comprehensive generic leadership development model that address all the needs of an organisation and that of all its people. Individual organisations should consider the local organisational philosophy and context; whilst recognising the individuals’ personal and distinctive needs or career transition, whilst taking cognisance of each person’s prior learning. It is those generic “adjustments” referenced by Nicholson (1984) that should be considered during the transition from clinician to leader, as individual effects, behaviours and dispositions will differ from person to person. That, according to Nicholson is the reason for this “individualistic focus” [as] the central premise of the theory that individual differences in the characteristics of people and the transitions they undergo mediate the relationships of “change vs stability” and “individual vs situational adjustment” (Nicholson, 1984, p. 172) which is suggested requires a better insight into the individual’s needs, from a developmental perspective.

It is also suggested that this approach will support “the thriving transition cycle” (Harris *et al*., 2012 p. 18) as it contributes to an expansion of Nicholson’s model (1988) and provides the framework for success for both managers and leaders in transition (Harris *et al*., 2012, p. 18). It could therefore be argued that a “transition develop model” based on the work of Nicholson and Harris *et al.* add to the discourse of management and leadership development (Harris *et al*., 2012; Nicholson, 1984; Nicholson and West, 1988).

The leadership development framework and the analysis of the data suggest that a foundation level development model for individual managers and leaders should be considered, not as a tick-box exercise, and not as a single method for all, but real education and development in the full sense of the word, based on the individual’s needs within the organisational context.

Considering the literature review and the data from this study, the following is offered as a definition for clinical leadership development.

Clinical leadership development is the education of clinical leaders in the art of understanding their own ability (the self), being present, engaging and listening; having an inquiring mind, exploring all the options from others (the other); and offering guidance that enables both “the self” and “the other” to benefit in terms of their own development, the people they lead, and ultimately resulting in improved patient outcomes.

Whilst every clinical leader may have different personal development needs, the development and identification of core elements of management for leaders are essential as the minimum requirements for any manager or leader to master. This will enable them to be good leaders and will empower them to develop themselves and the people they lead. Correctly modelled practical experience in leadership positions is rated highly in leadership learning, but essentially, the above should take place under supervision and with appropriate support (Hougaard *et al*., 2022; West, 2021; West *et al*., 2015; West and Bailey, 2019).

## Conclusion and implementation

West *et al.* (2015) confirmed that there is no best way to develop leaders, as it is always context sensitive, mostly influenced by the gap analysis and depending on nurturing a culture that enables staff to deliver continuous “improved high-quality, safe and compassionate care”. This influences how clinical leaders behave; the things they pay attention to, and how they develop themselves and others (Oliver and Memmott, 2006; Schein, 2004). The focus on development of clinical leaders should help them to be comfortable in making difficult decisions.

Creating multi-disciplinary teams across organisation boundaries and systems requires new ways of working, personal accountability, innovative ideas and system approaches for better patients’ outcomes. In the UK, as in the USA, there is “a renewed emphasis on accountability and managing unprofessional behaviours in the delivery of clinical care” (Bhardwaj, 2022).

Most participants in this study confirmed that they did not have appropriate management and/or leadership development to equip them in performing their duties to the best of their abilities. When implementing the leadership development framework, it is essential that a sound foundation is established. This starts with a value adding appraisal methodology (Carter, 2016; Kerr, 2018), which is essential as the primary source document for talent management, succession planning and an opportunity to have a wellbeing check-in with staff. It is a good process to re-enforce the organisational values and behaviours whilst defining how staff behaviours should demonstrate the values of an organisation. Dixon-Woods *et al.* (2013) and West (2021) contends that appraisals will help to understand the longer-term career aspirations of staff, whilst offering the support that may be required. Focusing on engagement, West *et al.* (2015), West (2021) confirms that regular compassionate conversations with staff are an essential part of being a compassionate leader.

It is essential for leaders to understand the impact of their behaviour, style or abilities on those around them. One consideration is the use of 360-degree feedback (Kerr, 2018).

The core principles of leadership, supporting “a higher quality of leadership training” and enabling a better “job role transition” based on the individual’s needs will enable and empower, which result in an improved ability to understand “the self” and lead “the other”.

The data in this study suggest that managers and leaders need to be education, developed and equipped, to enable to lead the people in their charge, to hold both challenging and compassionate conversations, as both are an art and does not come naturally to all. Managers and leaders should enable the people they lead to be the best they could be in a safe and just environment, without sacrificing accountability or performance. Thus, not stepping in and doing for “the other”, but rather support, empower and equip “the other”, to sustainably perform.
